# Overexpression of an Outer Membrane Protein Associated with Decreased Susceptibility to Carbapenems in *Proteus mirabilis*

**DOI:** 10.1371/journal.pone.0120395

**Published:** 2015-03-10

**Authors:** Yi-Lin Tsai, Min-Cheng Wang, Po-Ren Hsueh, Ming-Che Liu, Rouh-Mei Hu, Yue-Jin Wu, Shwu-Jen Liaw

**Affiliations:** 1 Department and Graduate Institute of Clinical Laboratory Sciences and Medical Biotechnology, College of Medicine, National Taiwan University, Taipei, Taiwan, Republic of China; 2 Departments of Laboratory Medicine and Internal Medicine, National Taiwan University Hospital, College of Medicine, National Taiwan University, Taipei, Taiwan, Republic of China; 3 Department of Biomedical Informatics, Asia University, Taichung, Taiwan, Republic of China; 4 Department of Applied Chemistry, National Chiao Tung University, Taiwan, Republic of China; University of Kentucky College of Medicine, UNITED STATES

## Abstract

*Proteus mirabilis* isolates commonly have decreased susceptibility to imipenem. Previously, we found *P. mirabilis hfq* mutant was more resistant to imipenem and an outer membrane protein (OMP) could be involved. Therefore, we investigated the role of this OMP in carbapenem susceptibility. By SDS-PAGE we found this OMP (named ImpR) was increased in *hfq* mutant and LC-MS/MS revealed it to be the homologue of *Salmonella* YbfM, which is a porin for chitobiose and subject to MicM (a small RNA) regulation. We demonstrated that ImpR overexpression resulted in increased carbapenem MICs in the laboratory strain and clinical isolates. Chitobiose induced expression of *chb* (a chitobiose utilization operon). Real-time RT-PCR and SDS-PAGE were performed to elucidate the relationship of *hfq, impR, chb* and MicM in *P. mirabilis*. We found MicM RNA was decreased in *hfq* mutant and *chbBC*-intergenic region (*chbBC*-IGR) overexpression strain (chbIGRov), while *impR* mRNA was increased in *hfq* mutant, *micM* mutant and chbIGRov strain. In addition, mutation of *hfq* or *micM* and overexpression of *chbBC*-IGR increased ImpR protein level. Accordingly, chitobiose made wild-type have higher levels of ImpR protein and are more resistant to carbapenems. Hfq- and MicM-complemented strains restored wild-type MICs. Mutation of both *impR* and *hfq* eliminated the increase in carbapenem MICs observed in *hfq* mutant and ImpR-complementation of *hfq/impR* double mutant resulted in MICs as *hfq* mutant, indicating that the ImpR-dependent decreased carbapenem susceptibility of *hfq* mutant. These indicate MicM was antisense to *impR* mRNA and was negatively-regulated by *chbBC*-IGR. Together, overexpression of ImpR contributed to the decreased carbapenem susceptibility in *P. mirabilis*.

## Introduction

*Proteus mirabilis* is an important pathogen of the urinary tract, especially in patients with indwelling urinary catheters [1]. Because of intrinsic resistance to polymyxin B [2] and production of enzymes, such as extended-spectrum β-lactamases (ESBLs) [3], carbapenemases [4] and AmpC [5], treatment of *P. mirabilis* infections could be difficult. Carbapenems are often the drugs of last resort for ESBL-producing organisms which are increasingly multi-drug resistant [6,7]. However, the emergence of carbapenem-resistant bacteria (CRB) jeopardizes the use of carbapenems [6,8]. In particular, *P. mirabilis* is intrinsically less susceptible to imipenem which is active for most enterobacteria [9,10].

Mechanisms of carbapenem resistance include target alterations, production of carbapenemases, efflux pumps and porin deficiency. Reduced expression of penicillin binding proteins (PBP) is associated with carbapenem resistance in *Acinetobacter* and *Proteus* [9,11]. A consistent number of acquired carbapenemases have been identified during the past few years, belonging to either metallo-β-lactamases or serine carbapenemases, and genes encoding these enzymes are associated with mobile genetic elements that allow their rapid dissemination in the clinical setting [6–8]. Overexpression of *Acinetobacter* AdeABC and *Pseudomonas* CzcCBA efflux pumps has been reported to be implicated in resistance to carbapenems [12,13]. Loss of porins engenders imipenem resistance in *Pseudomonas, Klebsiella* and *Acinetobacter* [13–15].

The study of carbapenem resistance in *P. mirabilis* is still lacking. Neuwirth et al. found reduced imipenem-affinity of PBP2 in two imipenem-insusceptible *P. mirabilis* isolates [9]. In addition, Tibbetts et al. first reported a carbapenem resistant *P. mirabilis* caused by the acquisition of *bla*_KPC-2_ [4]. *P. mirabilis* isolates exhibiting decreased susceptibility to imipenem also have been shown to carry a *bla*_VIM-1_ or a *bla*_NDM-1_ metallo-β-lactamase gene [16,17]. Although there is a report of imipenem resistance in a *P. mirabilis* strain associated with the loss of a 24 kDa OMP [18], no conclusion was drawn concerning *P. mirabilis* OMPs and carbapenem susceptibility.

Previously, we found Hfq is a pivotal coordinator for a diversity of regulatory circuits including surface components and virulence in *P. mirabilis* [19] and *hfq* mutant exhibited increased susceptibility to many antibiotics except imipenem (data not shown). Hfq is a posttranscriptional regulator that binds small RNAs (sRNAs) and mRNA and facilitates RNA-RNA interaction [20]. Numerous cellular processes, such as stress responses and OMP biogenesis are subject to the control of sRNAs and Hfq [21]. OMP analysis of *P. mirabilis hfq* mutant revealed a protein band (around 48 kDa) of increased intensity. We investigated the role of this OMP in carbapenem susceptibility and disclosed an Hfq-MicM (a sRNA) mediated process involved in decreased carbapenem susceptibility via upregulation of this OMP, named ImpR. This is a novel report elucidating how a sRNA-regulated OMP contributes to decreased susceptibility to carpapenems in *P. mirabilis*. The results indicate that an overexpressed OMP, neither a part of an efflux pump nor an OprD-like porin for drug entry, is associated with decreased susceptibility to carbapenems. This study also highlights the importance of sRNAs in drug susceptibility.

## Materials and Methods

### Bacterial strains, plasmids, reagents and culture conditions

The bacterial strains and plasmids used in this study are listed in Table 1. Bacteria were routinely cultured at 37°C in Luria-Bertani (LB) broth. The LSW^-^ agar [2] was used to prevent the phenotypic expression of swarming motility. Chitobiose, the β-1, 4-linked disaccharide of *N*-acetylglucosamine, was prepared using *Serratia marcescens* chitinase A [22].

**Table 1:** Bacterial strains and plasmids used in this study.

Strain or plasmid	Genotype or relevant phenotype	Source or reference
*P. mirabilis*		
wt	Wild-type N2; Tc^r^	[2]
hfq	wt derivative; *hfq*-knockout mutant; Km^r^	[19]
micM	wt derivative; *micM*-knockout mutant; Km^r^	This study
impR	wt derivative; *impR*-knockout mutant; Cm^r^	This study
hfq/impR	wt derivative; *hfq/impR*-knockout mutant; Km^r^ Cm^r^	This study
chbIGRov	wt containing pGEM-T Easy-*chbBC*-IGR; *chbBC*-IGR overexpressing strain; Amp^r^	This study
ImpRov	wt containing pGEM-T Easy-*impR*; *impR* overexpressing strain; Amp^r^	This study
hfqca	hfq mutant containing pGEM-T Easy-*hfq*; Hfq-complemented strain; Amp^r^	[19]
hfq/impRc	hfq/impR mutant containing pGEM-T Easy-*impR*; hfq/impR mutant complemented with ImpR; Amp^r^	This study
hfqca/impR	hfq/impR mutant containing pGEM-T Easy-*hfq*; hfq/impR mutant complemented with Hfq; Amp^r^	This study
micMc	micM mutant containing pGEM-T Easy-*micM*; MicM-complemented strain; Amp^r^	This study
*E. coli*		
DH5α	*fhuA2 lac(del)U169 phoA glnV44 Φ80' lacZ(del)M15 gyrA96 recA1 relA1 endA1 thi-1 hsdR17*	Invitrogen
S17–1 λ *pir*	λ *pir* lysogen of S17–1 [*thi pro hsdR* ^-^ *hsdM* ^+^ *recA* RP4 2-Tc::Mu-Km::Tn*7* (Tp^r^ Sm^r^)]; permissive host able to transfer suicide plasmids requiring the Pir protein by conjugation to recipient cells	[2]
Plasmids		
pGEM-T Easy	High-copy TA cloning vector; Amp^r^	Promega
pUT-Km1	Suicide plasmid requiring the Pir protein for replication and containing a mini-Tn*5* cassette containing Km^r^ gene	[2]
pACYC184	Low-copy cloning vector, P15A replicon; Cm^r^ Tet^r^	[2]
pGEM-T Easy-*chbBC*-IGR	pGEM-T Easy containing intact *chbBC*-IGR sequence; Amp^r^	This study
pGEM-T Easy-*impR* (pImpR)	pGEM-T Easy containing intact *impR* sequence including its ribosome binding site only for overexpression or its promoter for complementation; Amp^r^	This study
pACYC184-*chb-xylE*	*chb* reporter plasmid, pACYC184 containing intact *chb* promoter sequence before *xylE*; Cm^r^	This study
pGEM-T Easy-*micM*	pGEM-T Easy containing intact *micM* sequence including its promoter; Amp^r^	This study

### Construction of *P. mirabilis* mutants

Sequences flanking *micM* gene were amplified by PCR using the primer pairs micM-upF/XbaI-micM-upR and XbaI-micM-downF/micM-downR, respectively (Table 2) and cloned into pGEM-T Easy (Promega) to generate pGmicM-up and pGmicM-dn. pGmicM-up was digested with SalI/XbaI, and the *micM* upstream sequence-containing fragment was ligated to SalI/XbaI-digested pGmicM-dn to produce the pGmicM-updn plasmid, which contains the combined upstream and downstream sequences of *micM*. A Km^r^ cassette was inserted in the XbaI-digested pGmicM-updn plasmid to generate pGmicM-updn-Km. pGimpR-updn-Cm was constructed in a similar way except using primer pairs impR-upF/XbaI-impR-upR and XbaI-impR-downF/impR-downR and a Cm^r^ cassette for insertion. The DNA fragment containing the combined upstream and downstream sequence of *micM* or *impR* gene disrupted by Km^r^ or Cm^r^ cassette was cleaved from pGmicM-updn-Km or pGimpR-updn-Cm and ligated into SalI/SphI-cleaved pUT-Km1 to generate pUTmicM-Km and pUTimpR-Cm, respectively. Gene inactivation mutagenesis by homologous recombination and confirmation of mutants with double-crossover events were performed as described previously [2]. The *hfq*/*impR* double mutant (Km^r^ and Cm^r^) was constructed in a similar way using an existing *hfq* mutant (Km^r^). Those mutants were validated by sequencing and determining carbapenem MICs of mutants and their complemented strains to demonstrate the specificity of deletion.

**Table 2:** Primers used in this study.

Primers	Sequence (5’ to 3’)	Description
micM-upF	GAGATCCACACATTCAATC	For *micM* knockout
XbaI-micM-upR	TCTAGAAAGCCTCTGGTATCTAAAG	
XbaI-micM-downF	TCTAGACCTCTTAACGATAGAATAGC	For *micM* knockout
micM-downR	AGGCTGAAATGTATTCACC	
impR-upF	CTGGTGCTGAAGAGGATTTC	For *impR* knockout
XbaI-impR-upR	TCTAGATGCCTGGTTTAGCATGTTG	
XbaI-impR-downF	TCTAGAGTGATCATGCCATTTACG	For *impR* knockout
impR-downR	AAATAGACCACACTACGGG	
chbBC-IGR-overF	GGCCAAGAAGCGGATGTCG	For *chbBC*-IGR overexpression
chbBC-IGR-overR	TACCGTCATAAAGGCGGCG	
impR-overF	AGTCAAAGTCAAAGAGGAAAC	For *impR* overexpression
impR-overR	AATACACTTTATCCTTATTG	
micMc-F	TGATTCTACCATAGAACCATTTCC	For *micM* complementation
micMc-R	TGATATCGCCATTGAAAC	
chbre-F	GCATGCTGTAACGCGAAACAATGC	Amplification of *chb* promoter for reporter assay
chbre-R	CTGCAGAACCCTCTCAAATTGGCC	
impRrt-F	GAAAATGTCTTATGCTGAAG	For *impR* real-time RT-PCR
impRrt-R	CGTTAAAGGTAGAGTGGTG	
chbBC-IGRrt-F	ATATGGGAAAGTGGATGGATTAGG	For *chbBC*-IGR real-time RT-PCR
chbBC-IGRrt-R	ACCCTTAAAATGCCGCTAATTG	
micMrt-F	AAGAGGGCGGAGTGATGA	For *micM* real-time RT-PCR
micMrt-R	CGGCCAGTCAAAGAGGAATT	
gyrBrt-F	GACCCGTACGCTAAACAAC	Internal control for real-time RT-PCR
gyrBrt-R	AGAAATAACCGCAATCAGG	

For complementation of *micM* mutant, the fragment containing full-length *micM* was amplified by PCR using the primer pair, micMc-F/micMc-R, and cloned into pGEM-T Easy (Promega) to generate the plasmid, pmicM-com. The pmicM-com was transformed into the *micM* mutant to generate the MicM-complemented strain. The ImpR-complemented plasmid (pimpR-com) was constructed in a same way using the primer pair, impR-upF/impR-overR, to amplify the fragment containing full-length *impR*. pimpR-com and pGEM-T Easy-*hfq* [19] were transformed into hfq/impR double mutant, respectively, to generate the ImpR- and Hfq-complemented strains (hfq/impRc and hfqca/impR).

### Construction of *impR* and *chbBC*-IGR overexpressing strains

Full-length genes of *impR* and *chbBC*-IGR were amplified by PCR and cloned into pGEM-T Easy to generate pGimpR and pGchbBC-IGR, respectively. *impR* and *chbBC*-IGR are thus driven by the *lac* promoter in the pGEM-T Easy plasmid. The primers used in this study are listed in Table 2. pGimpR, and pGchbBC-IGR were then transformed into the wild-type *P. mirabilis* to generate the *impR* and *chbBC*-IGR overexpression strains. To study the effect of *impR* overexpression on the carbapenem susceptibility of clinical *P. mirabilis* isolates, pGimpR was also transformed into the clinical isolates.

### Minimum inhibitory concentration assay

The carbapenem MICs were determined by the broth microdilution method according to the guidelines of the Clinical and Laboratory Standards Institute (M07-A9) [23].

### Outer membrane protein analysis

Analysis of OMPs was carried out by SDS-PAGE. OMPs were prepared from bacteria grown overnight in LB according to the protocol described previously [24]. The OMP obtained was quantified by the Bio-Rad protein assay and adjusted to the same concentration before SDS-PAGE. We identified the OMP band that was significantly increased in *hfq* mutant, *micM* mutant, ImpRov (*impR*-overexpressing strain), and chbIGRov (*chbBC*-IGR-overexpressing strain) relative to wild-type and also this band of wild-type in the presence of chitobiose by liquid chromatography-tandem mass spectrometry (LC–MS/MS) using a hybrid dual-cell quadrupole linear ion trap (LTQ-Orbitrap Velos, Thermofisher Scientific) at Medical Center, College of Medicine, National Taiwan University.

### Reporter assay

The promoter region of the putative *chb* operon was amplified by the primer pair, chbre-F/chbre-R, (Table 2) and cloned into pGEM-T Easy to generate pGchbp. pGchbp was cut by SphI/PstI and the promoter-containing fragment was ligated with the *xylE* containg pACYC184 to construct the *chb-xylE* reporter plasmid. The overnight cultures of the wild-type transformed with the reporter plasmid (*chb-xylE*) were diluted 100 fold in the same medium with or without chitobiose and the XylE activity was measured as described previously [2] at time points indicated after incubation at 37°C.

### Real-time reverse transcription PCR (real-time RT-PCR)

Overnight LB cultures of wild-type and its derived strains (hfq, impR, micM, hfq/impR and chbIGRov) were diluted in LB broth to an optical density at 600 nm of 0.1, and grown overnight at 37°C adding chitobiose or not. Total RNA was extracted and real time RT-PCR was performed as described [2] to monitor the RNA levels of *impR, micM* and *chbBC*-IGR using primer pairs listed in Table 2. The RNA levels were normalized against the housekeeping gene, *gyrB*.

### Nucleotide sequence accession numbers

The nucleotide sequences of *P. mirabilis* N2 *impR* and *micM* genes have been deposited in GenBank under accession no. KM006423 and KM006424, respectively.

## Results

### Identification of ImpR, an OMP increased in *P. mirabilis hfq* mutant, contributing to decreased carbapenem susceptibility

**Fig 1:**
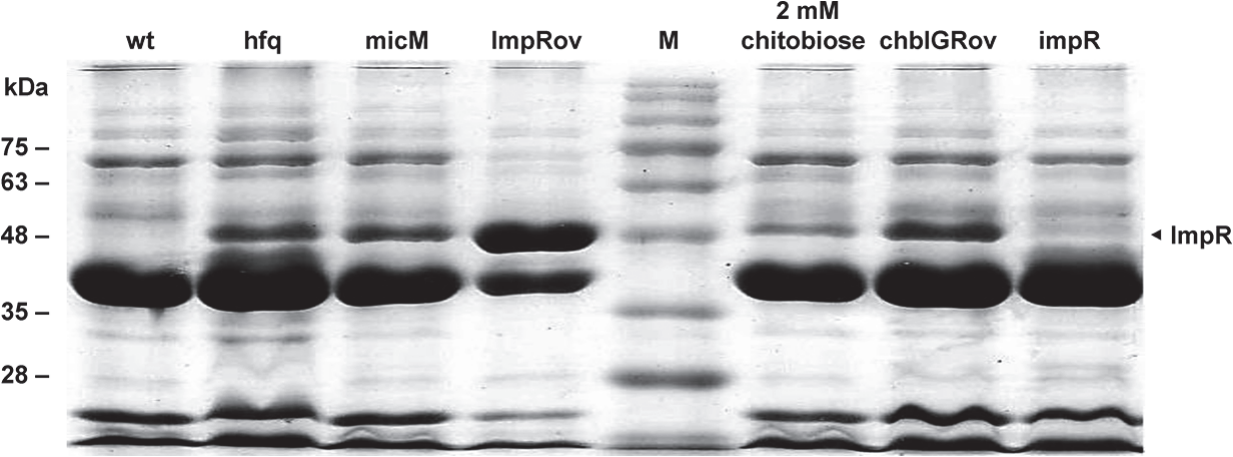
The SDS-PAGE profile of OMPs from overnight cultures of wild-type, its derivatives and wild-type treated with chitobiose. The representative result from three independent experiments is shown. The arrow indicates the band of ImpR. M, marker; wt, wild-type; hfq, *hfq* mutant; micM, *micM* mutant; ImpRov, ImpR-overexpressing strain; chbIGRov, *chbBC*-IGR-overexpressing strain; impR, *impR* mutant.

**Fig 2:**
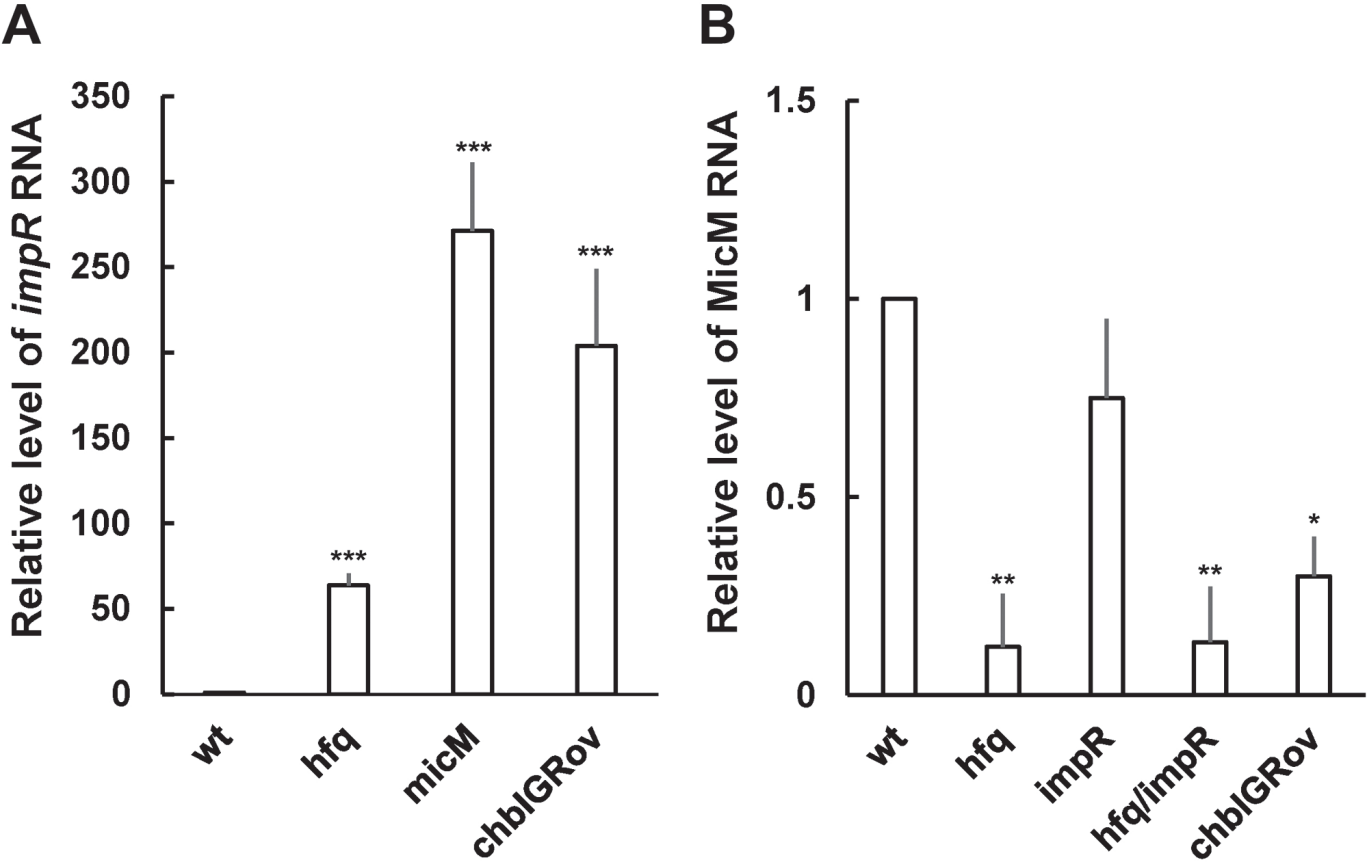
The RNA levels of *impR* (A) and MicM (B) in the wild-type *P. mirabilis* and its derived strains. The relative RNA levels of *impR* in the wild-type, mutants of *hfq* and *micM*, and chbIGRov were quantified by real-time RT-PCR. The RNA was prepared using overnight bacterial cultures. The relative RNA levels of MicM in the wild-type, mutants of *hfq, impR* and *hfq/impR*, and chbIGRov were also determined in the same way. The expression level for the wild-type cells was set at 1. The data represent the averages of three independent experiments with standard deviations. Significant difference from the wild-type was indicated with the asterisk (*, *P*<0.05; **, *P*<0.01; ***, *P*<0.001 by Student’s t-test analysis). wt, wild-type; hfq, *hfq* mutant; impR, *impR* mutant; hfq/impR, *hfq* and *impR* double mutant; chbIGRov, *chbBC*-IGR overexpressing strain; micM, *micM* mutant.

Previously, we found *P. mirabilis hfq* mutant exhibited increased resistance to imipenem. SDS-PAGE analysis revealed an OMP about 48 kDa was increased in *hfq* mutant (Fig. 1). We confirmed that imipenem MIC of *hfq* mutant was increased 4-fold compared to the wild-type (Table 3) and identified the OMP (named ImpR) as YbfM protein (an OMP for chitobiose utilization) homologue of *Salmonella* [25] by LC-MS/MS. Real-time RT-PCR also showed a much higher *impR* mRNA level in *hfq* mutant relative to the wild-type which is almost silent (Fig. 2A). To further investigate the role of ImpR in imipenem susceptibility, we constructed the ImpRov strain (Table 1) by transforming the ImpR-overexpressing plasmid (pImpR) to the wild-type and found the ImpRov strain had a 4-fold higher imipenem MIC level than the vector only control (Table 3). SDS-PAGE analysis revealed a band (about 48 kDa) of increased intensity in the ImpRov strain (Fig. 1) and the band was identified as *Salmonella* YbfM homologue by LC-MS/MS.

**Table 3:** MICs of imipenem, meropenem and ertapenem for *P. mirabilis* N2 and its derived strains.

	MIC (μg/ml)		
Strain	imipenem	meropenem	ertapenem
wt	1	0.06	0.015
hfq	4	0.24	0.06
hfq-vector	4	0.24	0.06
hfqca	1	0.06	0.015
wt-vector	1	0.06	0.015
ImpRov	4	0.24	0.06
wt-chitobiose*	4	0.24	0.03
impR	1	0.06	0.015
impRc	4	0.24	0.06
hfq/impR	1	0.06	0.015
hfq/impR-vector	1	0.06	0.015
hfq/impRc	4	0.24	0.06
hfqca/impR	1	0.06	0.015
micM	4	0.24	0.06
micM-vectormicMc	41	0.240.06	0.060.015
chbIGRov	4	0.24	0.03

*, 2 mM; wt, wild-type; hfq, *hfq* mutant; hfqca, Hfq-complemented strain; ImpRov, ImpR-overexpressing strain; impR, *impR* mutant; impRc, *impR* mutant complemented with ImpR; hfq/impR, *hfq/impR* double mutant; hfq/impRc, hfq/impR complemented with ImpR; hfqca/impR, hfq/impR complemented with Hfq; micM, *micM* mutant; micMc, MicM-complemented strain; chbIGRov, *chbBC* intergenic region-overexpressing strain.

**Fig 3:**
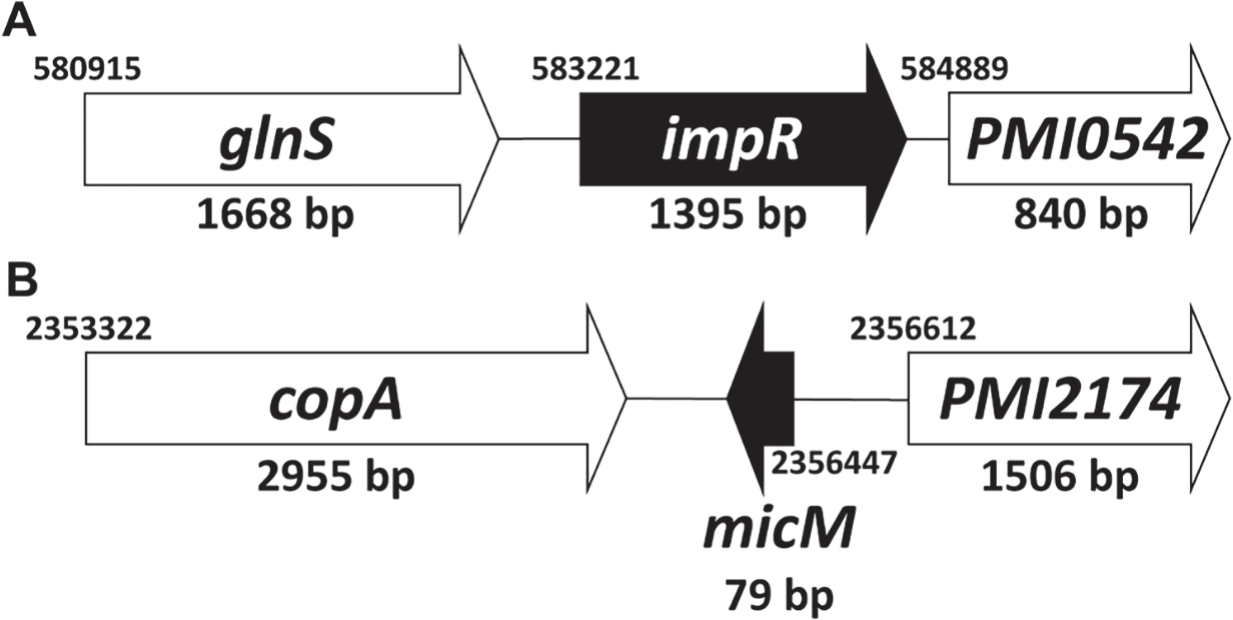
Genomic location of *impR* (A) and *micM* (B) in *P. mirabilis*. The number indicates the start nucleotide number of each gene in the genome and the size (bp) of each gene is indicated.

The *impR* gene was identified at nt 583221 to 584615 in the genome of *P. mirabilis* strain HI4320. It is located in the *glnS-impR-PMI0542* cluster as shown in Fig. 3A. The ImpR protein consists of 464 amino acids and shares 64% sequence identity and 77% similarity with its homologue, YbfM of *Salmonella*. Using primers annealing to conserved sequences, we cloned and sequenced the fragment containing *impR* and upstream of *impR* in *P. mirabilis* N2. The amino acids of N2 ImpR were 100% and 99% identical to those of *P. mirabilis* HI4320/*P. mirabilis* BB2000 and *P. mirabilis* WGLW4, respectively.

### Searching for a small RNA regulating expression of *impR*

It has been known that YbfM is a porin required for uptake of chitobiose [25–27]. In the absence of chitobiose (inducer), *ybfM* is kept silent by the action of a constitutively-made sRNA, MicM, which pairs with the 5’ UTR of *ybfM* mRNA [25–27]. Silencing is relieved in the presence of inducer due to accumulation of an RNA that pairs with MicM, thus promoting MicM degradation. The anti-MicM RNA is from an intergenic region (between *chbB* and *chbC*, called *chbBC*-IGR) of the *chb* operon (*chbBCARFG*), which contains genes for chitobiose utilization and whose transcription is activated in the presence of chitobiose [28]. We first searched *P. mirabilis* MicM homologue in the website, http://bac-srna.org/BSRD/index.jsp#, and located it in genome of *P. mirabilis* strain HI4320. Although the *ybaK-micM-ybaP* region is well conserved in many enterobacteria, we found *P. mirabilis* MicM is between *copA* gene and *PMI2174* (Fig. 3B). We also found the existence of *chb* operon homologue in *P. mirabilis* strain HI4320. We cloned and sequenced the fragment containing *micM* and upstream of *micM* in *P. mirabilis* N2. The nucleotide sequences of *micM* were 100% identical to those of *P. mirabilis* HI4320.

Sequence analysis revealed ‘5’UGAAAAAUUCCUCUUUGACUGG’ could be the site for MicM to bind with *impR* mRNA and the anti-MicM region of the *chb* mRNA.

**Fig 4:**
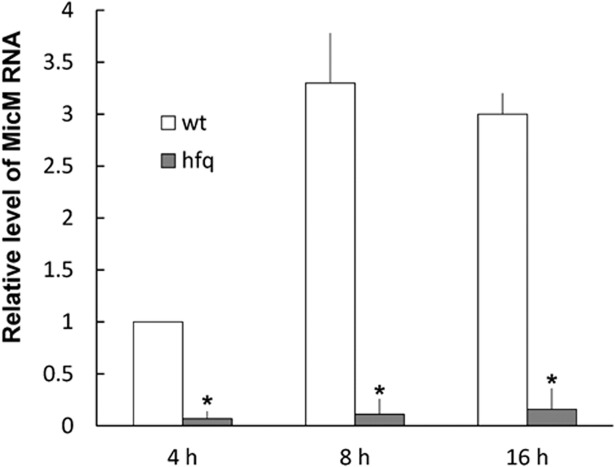
The expression of MicM in the wild-type and *hfq* mutant. The RNA amount of MicM in the wild-type or *hfq* mutant was quantified by real-time RT-PCR. Overnight bacterial cultures were diluted to OD_600_ of 0.1 and incubated for 4 h, 8 h and 16 h before total RNA was prepared. The expression level for the wild-type cells at 4 h was set at 1. The data represent the averages of three independent experiments with standard deviations. Significant difference from the wild-type at different time point was indicated with the asterisk (*, *P*<0.01 by Student’s *t*-test analysis). wt, wild-type; hfq, *hfq* mutant.

**Fig 5:**
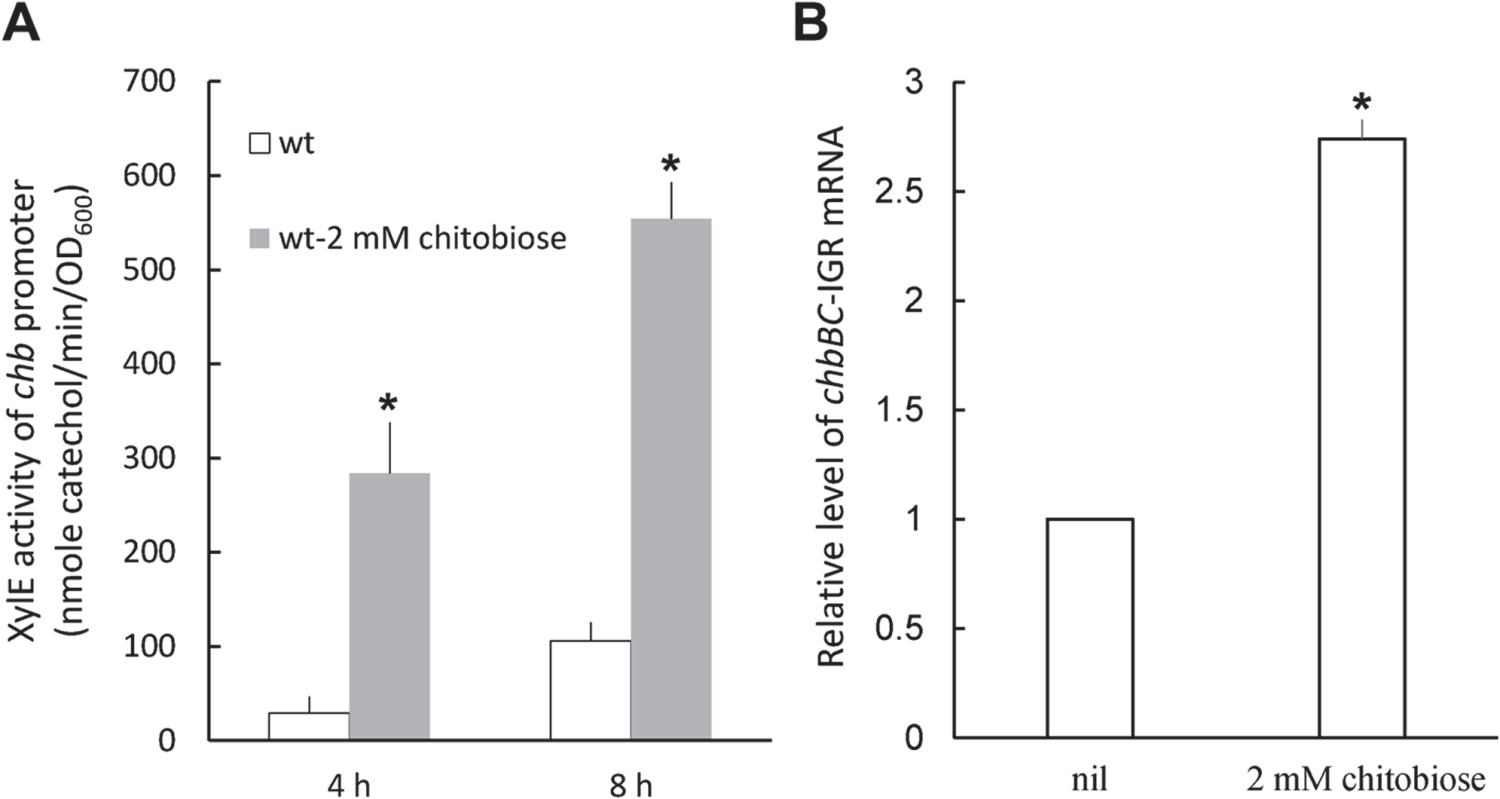
(A) Chitobiose induced *chb* promoter activity. The *xylE* activity in the *chb-xylE* reporter plasmid-transformed wild-type *P. mirabilis* in the presence or absence of 2 mM chitobiose was measured after induction for 4 h and 8 h. **(B) Chitobiose induced expression of *chb* mRNA.** The RNA of the wild-type was prepared using overnight bacterial cultures and relative *chb* mRNA amount was quantified by real-time RT-PCR using primers for *chbBC*-IGR. The expression level of control cells without chitobiose (nil) was set at 1. The data represent the averages of three independent experiments with standard deviations. Significant difference from the no chitobiose control at different time point was indicated with the asterisk (*, *P*<0.01 by Student’s *t*-test analysis). wt, wild-type.

Knowing the existence of MicM and *chb* operon in *P. mirabilis*, we first performed real-time RT-PCR to assess the MicM level in wild-type and *hfq* mutant. Fig. 4 indicated MicM was constitutively expressed and subjected to the positive control of Hfq. Constitutive expression of MicM could explain the very low level of ImpR in wild-type (Fig. 2). In addition, Fig. 5 showed that chitobiose can induce *chb* promoter activity and consequently lead to the increased *chb* mRNA (*chbBC*-IGR) level.

### Characterization of MicM-mediated regulation of *impR*

To know if the regulation of Hfq-dependent MicM by *chb* mRNA and *impR* by MicM exists in *P. mirabilis*, we constructed single mutants of *impR* and MicM, *hfq*/*impR* double mutant and *chbBC*-IGR overexpressing strain (chbIGRov). Real-time RT-PCR was performed to clarify the relationship of *chb*, MicM, *hfq* and *impR*. Fig. 2B showed *impR* mutation didn’t affect MicM level, whereas *hfq*/*impR* double mutation led to a significantly decreased level of MicM. The result confirmed MicM is positively regulated by Hfq. In addition, chbIGRov strain exhibited a significantly decreased MicM level. Moreover, either overexpression of *chbBC*-IGR or mutation in *hfq* or *micM* resulted in a high level of *impR* mRNA. OMP analysis also revealed that *chbBC*-IGR overexpression strain (chbIGRov), *micM* mutant and *hfq* mutant all had an increased level of ImpR protein (Fig. 1). In accordance with the finding that chitobiose can induce expression of *chb* operon (Fig. 5), ImpR protein was increased in wild-type treated with chitobiose (Fig. 1). Together, these results indicated antisense regulation of *impR* mRNA by MicM, Hfq-dependent expression of MicM and negative regulation of MicM by *chb* operon in response to chitobiose.

### Significance of MicM-mediated regulation of *impR* in carbapenem susceptibility of *P. mirabilis*

MIC assay was conducted to clarify the significance of MicM-mediated regulation of *impR* in carbapenem susceptibility of *P. mirabilis*. Firstly, carbapenem MICs were determined in wild-type, derived mutants and the complemented strains to validate that mutation of *hfq, impR* or *micM* was involved in carbapenem susceptibility. We noticed that *impR* mutant had wild-type MIC (Table 3). Hfq- and MicM-complemented strain restored wild-type carbapenem MICs. hfq/impR double mutant complemented with ImpR had the same carbapenem MICs as *hfq* mutant and exhibited wild-type MICs when complemented with Hfq (Table 3). The finding that MICs of *impR* single and *hfq/impR* double mutants were the same as wild-type (Table 3) indicated the low expression of *impR* in wild-type and the ImpR-dependent carbapenem-resistance for *hfq* mutant. Besides, chitobiose (the inducer for *chb* operon) and overexpression of *chbBC*-IGR caused a 4-fold increase in imipenem MICs compared to the wild-type (Table 3). Accordingly, *micM* mutant had a 4-fold increase in imipenem MICs (Table 3). It is worth noting that mutation of *micM* or *hfq*, overexpression of ImpR or *chbBC*-IGR, and chitobiose also increased meropenem and ertapenem MICs to 4 and 2–4 fold, respectively (Table 3).

To further demonstrate the significance of ImpR in carbapenem susceptibility of *P. mirabilis*, we introduced the ImpR-overexpressing plasmid into clinical isolates of *P. mirabilis* and MICs were determined. Table 4 showed that clinical isolates bearing the ImpR-overexpressing plasmid exhibited 4–8 fold increase in imipenem and meropenem MICs relative to the vector control. We also found that MICs of ertapenem were increased 2–4 fold (Table 4).

**Table 4:** MICs of imipenem, meropenem, and ertapenem for *P. mirabilis* clinical isolates transformed with the ImpR-overexpressing plasmid.

	MIC (μg/ml)		
Clinical isolate		imipenem	meropenem	ertapenem
1	vector	1	0.06	0.015
	pImpR	4	0.25	0.06
2	vector	1	0.06	0.03
	pImpR	4	0.25	0.06
3	vector	1	0.06	0.06
	pImpR	4	0.25	0.25
4	vector	2	0.06	0.03
	pImpR	8	0.25	0.12
5	vector	1	0.12	0.06
	pImpR	4	0.48	0.12
6	vector	1	0.03	0.03
	pImpR	8	0.25	0.12

## Discussion

It is imperative to investigate carbapenem resistance to escape the public health crisis caused by CRB. Although *P. mirabilis* is still susceptible to meropenem and ertapenem, the bacterium is known to be intrinsically less susceptible to imipenem [9,10]. Previously, we accidentally found that decreased imipenem susceptibility of *P. mirabilis hfq* mutant was associated with an OMP. In this study, we described the role of the OMP (ImpR) in decreased imipenem susceptibility, also meropenem and ertapenem. The phenotype was mediated through an Hfq-regulated sRNA, MicM, in a process involving *chb* operon in response to chitobiose. Several lines of evidence support the notion. First, increased ImpR, either by mutation of *hfq* or *micM* (MicM is antisense to *impR* mRNA) or overexpression of ImpR rendered carbapenem MICs to increase 4-fold (Table 3). Second, levels of *impR* mRNA and ImpR protein were increased but MicM RNAs were decreased in *hfq* mutant (Figs. 1 and 2), consistent with that Hfq-dependent MicM is antisense to *impR* mRNA. Third, chitobiose induced expression of ImpR protein (Fig. 1) and *chb* mRNAs (Fig. 5), antisense to MicM. chbIGRov strain displayed a decrease in MicM RNA level (Fig. 2) but an increase in expression of *impR* (Figs. 1 and 2). Both the presence of chitobiose and overexpression of *chbBC*-IGR resulted in increased carbapenem MICs (Table 3). Fourth, *hfq*/*impR* double mutation abolished the increased carbapenem MICs of *hfq* mutant (Table 3), indicating decreased carbapenem suaceptibility in *hfq* mutant depends on the ImpR protein. Fifth, the ImpR- overexpressing plasmid made the clinical isolates more resistant than its parent (Table 4).

Bioinformatic analysis revealed ImpR could be a porin of the OprD superfamily, such as *E. coli* Chip, *Salmonella* YbfM and *Pseudomonas* OprD [13,15,25,26], instead of OMPs of the RND efflux pumps, such as TolC [31]. A high glycine content and absence of cysteine residues also indicated ImpR was typical of a gram-negative bacterial porin [32]. In *Pseudomonas*, OprD loss usually causes a 4 to16-fold increase in MICs of carbapenems [13]. We found *P. mirabilis* ImpR is probably involved in the uptake of chitobiose because both *impR* mRNA (Fig. 2) and protein levels (Fig. 1) were induced by overexpression of *chbBC*-IGR (i.e. the presence of chitobiose). In addition to probably serving as a porin as Chip or YbfM for growing on chitobiose [25,27,33], ImpR also contributed to carbapenem susceptibility when overexpressed. Until now the role of Chip and YbfM in drug susceptibility has not been reported and we found the ImpR-overexpressing plasmid failed to affect carbapenem susceptibility in *E. coli* (data not shown), suggesting the uniqueness of ImpR overexpression in *P. mirabilis*. Mutation of *impR* did not affect carbapenem MICs and ImpR overexpression resulted in an increase in carbapenem MICs instead of a decrease of MICs in overexpression of the porin for carbapenem entry in other bacteria [13–15]. Low expression of ImpR resulting from antisense action of constitutively-expressed MicM in wild-type may explain why *impR* mutant has wild-type carbapenem MICs. A pump inhibitor, carbonyl cyanide m-chlorophenyl hydrazone, has no effect on carbapenem MICs of ImpRov (data not shown), indicating ImpR not a part of proton-motive pumps.

Our unpublished data showed that RpoE was up-regulated on ImpR overproduction. In this regard, RpoE overexpression has been shown to cause remarkable resistance to the β-lactam antibiotics [30] that cause cell envelope stress by inhibiting peptidoglycan biosynthesis. Accordingly, stress responses have been linked to the development of antimicrobial resistance in Gram-negative bacteria [34]. Oxidative stress is also an end product of antimicrobial exposure. Therefore, the oxidative stress response is expected to promote resistance to antimicrobials [34]. It is possible that the RpoE regulon involved in combating with either envelope or oxidative stresses [29] may contribute to the decreased carbapenem susceptibility in *P. mirabilis*. Further studies are needed to disclose the mystery.

It has been reported that more attention should be devoted to the mechanisms of low-level resistance in microorganisms, as they can serve as stepping stones to develop high level, clinically relevant resistance [35]. In this study, we found ImpR contributes to decreased susceptibilities (low-level resistance) of carbapenems. What is the importance of ImpR in carbapenem susceptibility in the real world? First, cAMP has been shown to inhibit expression of *hfq* [36]. In this regard, low level of glucose in urine, a condition of high cAMP level, may lead to repression of Hfq, thus increased ImpR and subsequently decreased carbapenem susceptibility. Second, the presence of chitobiose in urine may also increase the level of ImpR. Chitobiose, the β-1,4-linked disaccharide of *N*-acetylglucosamine, is the major degradation product of chitin, which constitutes the second-most abundant organic polymer in nature after cellulose [33]. Third, we have found several imipenem-resistant clinical isolates of *P. mirabilis* whose expression of ImpR is higher than the susceptible ones. In addition, we can not rule out the alterations in the regulatory elements of *impR, hfq, micM* and *chbBC*-IGR in the natural environment, which may lead to upregulation of ImpR and thus decreased carbapenem susceptibility. With regard to intrinsic resistance of carbapenems in *P. mirabilis*, we have found an OMP mutant exhibited increased (8 fold) susceptibility to imipenem. Villar et al. also found decreased expression of an OMP in *P. mirabilis* was involved in increased susceptibility to imipenem and meropenem [10]. Characterization of the OMP has been underway.

**Fig 6:**
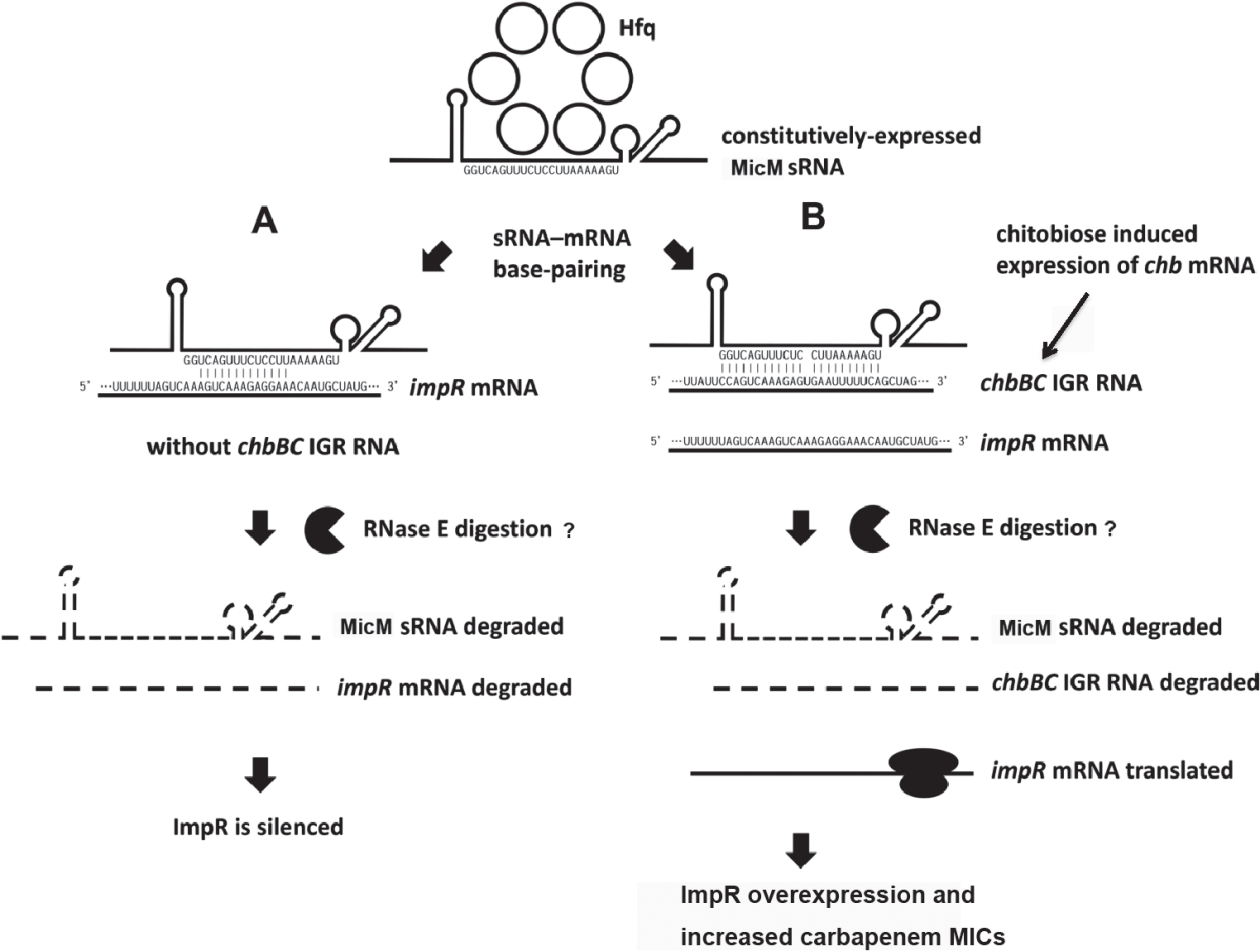
Model for ImpR regulation by MicM sRNA. (A) In the absence of chitobiose, the expression of the ImpR porin is silenced at the post-transcriptional level by pairing of MicM sRNA with the 5’ UTR of *impR* mRNA, promoting cleavage of the *impR* mRNA by a ribonuclease (RNase E?). At the same time the *chb* operon is transcriptionally repressed. (B) In the presence of chitobiose, an inducer to activate transcription of the *chb* operon, processing of the *chb* transcript releases *chbBC* IGR RNA. This RNA base-pairs with MicM making it susceptible to the action of a ribonuclease (RNase E?). The drop in MicM levels relieves *impR* repression leading to a burst of ImpR translation. ImpR assembles in the outer membrane resulting in increased carbapenem MICs. MicM is an Hfq-dependent and constitutively-expressed sRNA.

In this work, for the first time, we described the role of an OMP (ImpR) in decreased carbapenem susceptibility and its regulation by a sRNA (MicM) involving the *chb* operon in *P. mirabilis* (summarized in Fig. 6). These data suggest that upregulation of ImpR can make *P. mirabilis* become more resistant to carbapenem treatment, in contrast to down-regulation of OprD in *P. aeruginosa* [13,15]. The decreased carbapenem susceptibility incurred by increased ImpR has implications in carbapenem therapy against urosepsis caused by *P. mirabilis*. Clinicians should keep in mind about this acquired low-level resistance of carbapenems in the clinical setting.
